# Fistula Closure Using a Vastus Lateralis Skin Valve for Esophagobronchial Fistula Occurring During Preoperative Chemotherapy for Lung Cancer: A Case Report

**DOI:** 10.7759/cureus.59666

**Published:** 2024-05-05

**Authors:** Ryusei Yoshino, Masaki Nakatsubo, Nanami Ujiie, Masahiro Kitada

**Affiliations:** 1 Thoracic Surgery and Breast Surgery, Asahikawa Medical University Hospital, Asahikawa, JPN

**Keywords:** pembrolizumab, neoadjuvant chemotherapy, lung cancer, esophagorespiratory fistula, esophagobronchial fistula

## Abstract

An esophagobronchial fistula, an abnormal passageway formed between the esophagus and bronchus, can cause severe respiratory symptoms. This fistula is a complication that can occur during chemoradiotherapy for esophageal and lung cancers; however, to our knowledge, no esophagobronchial fistulas during preoperative chemotherapy for lung cancer have been reported. The patient was a 55-year-old man whose chest computed tomography (CT) revealed a mass on the dorsal bronchus and right side of the esophagus. A transesophageal needle biopsy confirmed the diagnosis of lung adenocarcinoma, and preoperative chemotherapy, which included pembrolizumab, was administered. One week after the first course of chemotherapy, the patient developed a severe cough after drinking water. Chest CT revealed an esophagobronchial fistula, which prompted the discontinuation of the preoperative chemotherapy. Subsequent conservative treatment resulted in no improvement, and the patient was referred to our department. One month thereafter, a two-stage reconstruction of the esophagus was performed via the posterior sternal route. The resected specimen showed no residual tumor in the lungs, and the treatment was determined to result in a complete pathological response. The patient is currently undergoing maintenance therapy with pembrolizumab as a single agent. This is a rare case of esophagobronchial fistula identified during preoperative chemotherapy that included pembrolizumab for lung cancer. In addition to suturing the fistula, filling it with a distal hyoid valve was effective in treating the esophagobronchial fistula.

## Introduction

An esophagobronchial fistula is an abnormal passageway formed between the esophagus and bronchus. In such instances, digestive fluids may enter the bronchus and lungs, causing respiratory symptoms. Although no standardized treatment is available for this disease, placement of esophageal or tracheal stents and endoscopic clipping may be considered [1,2]. Nevertheless, esophagobronchial fistulas are often refractory. In some cases, surgical closure of the fistula using a myocutaneous valve may be the treatment of choice; however, the prognosis is poor, and the choice is sometimes difficult.

Notably, reports on the closure of fistulas that are a postoperative complication of esophageal cancer or serious complication that can occur during chemoradiation therapy for advanced lung cancer are limited [3]. Herein, we report an esophagobronchial fistula identified during preoperative chemotherapy for lung cancer, in which two-stage esophageal reconstruction in addition to fistula closure using a pectoralis major valve was performed surgically. We believe this case is worth reporting because, to our knowledge, no such case has been reported before.

## Case presentation

The patient was a 55-year-old male whose height, weight, and body mass index were 177 cm, 45 kg, and 14.4 kg/m2, respectively. He had a history of chronic obstructive pulmonary disease but no history of oral medication. He was a current smoker of 20 cigarettes/day (Brinkman Index: 660) since the age of 22 years and had no history of alcohol consumption. Five months prior, he experienced back pain in his right chest, which prompted a thorough examination.

**Figure 1:**
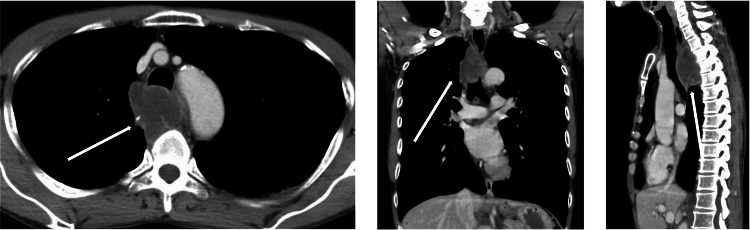
Chest computed tomography (CT) findings A mass is noted on the dorsal bronchus and right side of the esophagus before preoperative chemotherapy.

Chest radiography and computed tomography (CT) revealed a mass on the dorsal bronchus and right side of the esophagus (Figure 1). Transbronchial needle biopsy via bronchoscopy was performed; however, the patient was diagnosed with lung adenocarcinoma based on transesophageal needle biopsy results via upper gastrointestinal endoscopy. The immunohistochemistry results were as follows: TTF1 (-), p40 (-), CD56 (-), CK7 (+), CK20 (-), and PD-L1 > 75%. The Oncomine test results were negative for all mutations. Two months after the first visit, the patient was administered preoperative chemotherapy (cisplatin, pemetrexed, and pembrolizumab) because of progressive pain upon swallowing and esophageal stricture over time without evidence of distant metastasis in other organs. One week after the first course of preoperative chemotherapy, the patient developed a severe cough after drinking water, and a chest CT revealed an esophagobronchial fistula. Consequently, preoperative chemotherapy was stopped, and the patient fasted for approximately one month in anticipation of spontaneous closure of the fistula. However, no change in the condition was observed.

**Figure 2:**
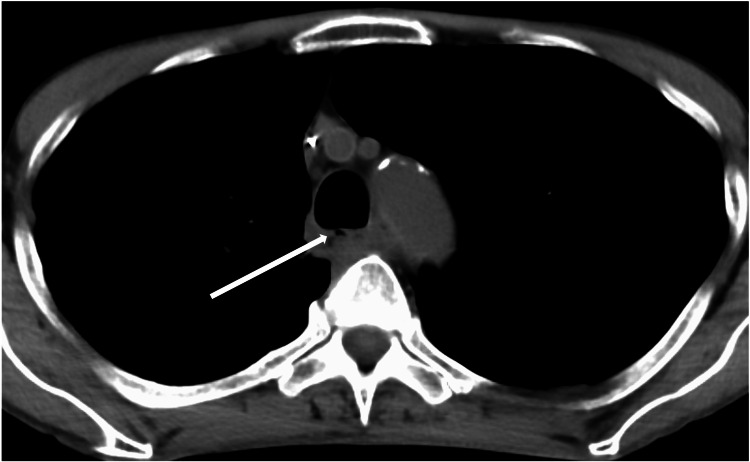
Chest computed tomography (CT) findings A small mass shadow is observed in the upper lobe of the right lung, and a fistula is observed between the right main bronchus and upper thoracic esophagus.

**Figure 3:**
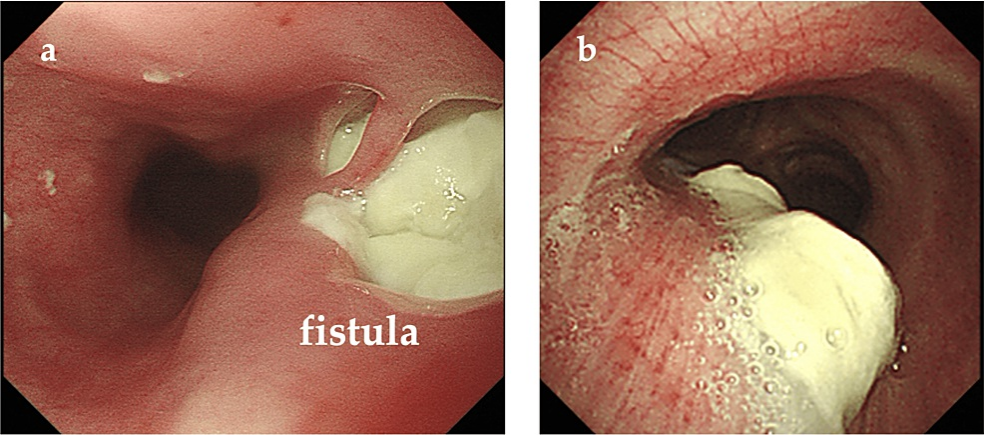
Bronchoscopy (A) and upper gastrointestinal endoscopy findings (B) (A) A 2-cm fistula is observed in the lumen of the right main bronchus, approximately 14 cm from the glottis. (B) A 2-cm fistula is observed in the upper esophagus, slightly above the tracheal bifurcation.

The patient was then referred to our department to undergo surgical treatment, and a thorough examination was performed to determine the patient’s condition. The blood analysis results were as follows: white blood cell count = 10,580/μL (neutrophil, 82.0%); hemoglobin level = 9.1 g/dL; platelet count = 32.3 × 104/μL; total protein level = 5.4 g/dL; albumin level = 2.1 g/dL; blood urea nitrogen level = 14.5 mg/dL; creatinine level = 0.54 mg/dL; sodium level = 134 mmol/L; potassium level = 4.1 mmol/L; chlorine level = 100 mmol/; blood sugar level = 130 mg/dL; and C-reactive protein level = 10.04 mg/dL. Electrocardiography showed no abnormalities and respiratory function tests revealed no restrictive or obstructive ventilatory dysfunction. Echocardiography revealed no evidence of valvular disease, and the ejection fraction was 60%. A chest CT showed a small mass in the upper lobe of the right lung and fistula formation between the right main bronchus and upper thoracic esophagus (Figure 2). There was no evidence of pneumonia. Bronchoscopy revealed a 2-cm fistula in the lumen of the right main bronchus, approximately 14 cm from the glottis, and a white necrotic material-like epithelium was observed from the fistula (Figure 3A). Upper gastrointestinal endoscopy revealed a 2-cm fistula in the upper esophagus, slightly above the tracheal bifurcation (Figure 3B). Based on these findings, the preoperative diagnosis was esophagobronchial fistula. After joint consultation with respiratory surgery, respiratory medicine, esophageal surgery, gastroenterology, and plastic surgery departments, bronchopleural fistula closure with a two-stage esophageal reconstruction surgery was performed.

**Figure 4:**
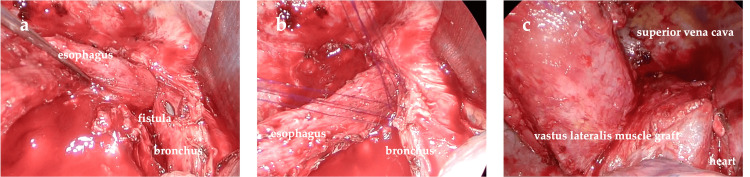
Intraoperative findings (A) The bronchus shows a slit fistula on the right lateral side from the membranous part. (B) The fistula is directly sutured to the surrounding tissue with five stitches using 4-0 polydioxanone (PDS) II sutures. (C) The fistula closure is coated with fibrin glue, and the stromal valves are suture-ligated to the surrounding tissue.

**Figure 5:**
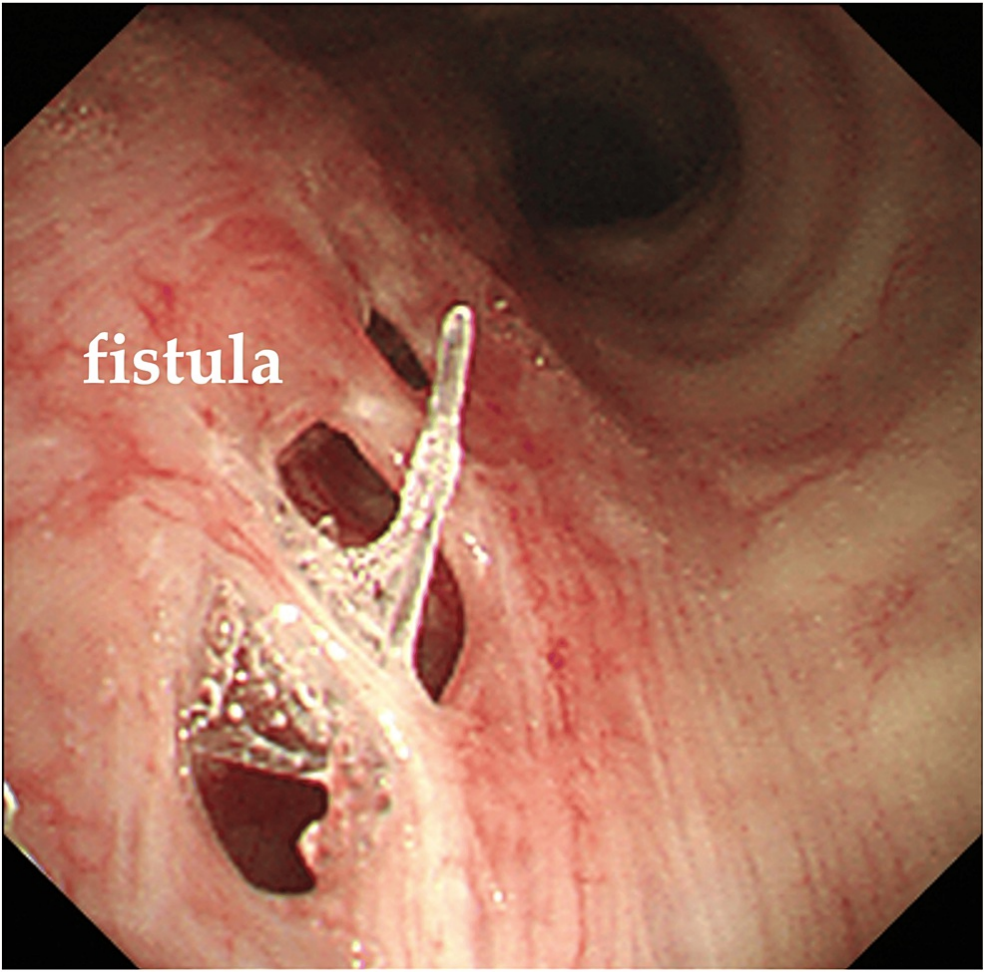
Postoperative bronchoscopy findings A fistula is observed at the same site noted preoperatively, although no white necrotic material-like epithelium is observed in the fistula.

The patient was placed in the left lateral recumbent position under general anesthesia with isolated lung ventilation. First, a J-shaped incision was made on the lateral side, and the subcutaneous tissue was dissected. The border of the vastus lateralis muscle was then reached, and a stemmed vastus lateralis valve was created. The fourth and fifth ribs were resected, and the thoracic cavity was reached. Overall, the adhesions, particularly those on the mediastinal side, were strong. Partial resection of the lung, including the tumor, was performed. Subtotal resection of the esophagus was performed; the cephalic side was resected at the apex of the lung, and the caudal side was resected by cutting at the level of the supradiaphragm. The esophagobronchial fistula, which was a slit-like fistula in the membranous part to the right lateral side, was reached, and five stitches were sutured directly to the surrounding tissue using 4-0 polydioxanone (PDS) II sutures (Figures 4A, 4B). The fistula closure was then filled with a previously harvested stapedial dorsalis valve. At that time, fibrin glue was applied to the fistula closure, and the sternohyoid valve was suture-ligated with the surrounding tissue (Figure 4C). Finally, a 24 Fr thoracic drain was inserted, and the chest was closed. The patient’s position was changed to supine, and a transverse incision was made on the left side of the neck to expose the cervical esophagus near the internal jugular vein and trachea. Subsequently, the remaining esophagus was pulled up from the thoracic cavity to create a stoma. The abdomen was opened through a 10-cm median incision above and below the umbilicus, and the jejunum was raised to create a Witzel jejunostomy (a 9 Fr jejunostomy catheter was used). The operative time was five hours and 33 minutes, and the blood loss was 150 mL. The patient’s thoracic drain was air leak-negative and fluctuation-positive postoperatively, and it was carefully removed on the 8th postoperative day. The seroma at the valve wound was drained as required. Postoperative bronchoscopy revealed a fistula at the same site noted preoperatively, and no white necrotic material-like epithelium was observed in the fistula (Figure 5). In addition, what appeared to be a filled vastus lateralis skin valve was observed in the dorsal aspect of the fistula.

One month postoperatively, the patient underwent a two-stage esophageal reconstruction (anastomosis of the cervical esophagogastric tube of the posterior sternal route) by an esophageal surgeon. A small 7-cm incision was made in the midline below the xiphoid process, and the small mesh was removed. However, the transverse colon and large mesh adhered around the jejunostomy site. Therefore, the incision was extended above the umbilicus, and a laparotomy was performed. The left gastric vena cava, gastrosplenic mesentery, and short gastric arteriovenous veins were excised. The left gastric vein and artery were dissected while preserving the collateral hepatic arteries. The periesophageal hiatus was dissected, the stomach was elevated, and the abdominal cavity was washed. Subsequently, the right gastric artery was dissected at the end of the third gastric branch, and a 3.5-cm wide gastric tube was created using Signia/purple. After administration of indocyanine green, blood flow in the gastroduodenal tube was confirmed using a PDE-neo camera (Hamamatsu Photonics, Shizuoka, Japan); a posterior sternal route was created, and the gastroduodenal tube was raised. The excess esophagus and gastrointestinal tube were resected, and the esophagogastric tube end was sutured with a full external triangular anastomosis using Signia/purple. A large gastrointestinal tract mesh was sutured to cover the anastomosis site. The operative time was five hours and 22 minutes, and the volume of blood loss was 98 mL. Enteral nutrition was initiated the following day. The patient resumed drinking water on the 6th postoperative day and eating on the 7th postoperative day; he was discharged from the hospital on the 15th postoperative day.

The postoperative resection specimen showed no residual tumor in the lungs; thus, the treatment was determined to result in complete remission. Therefore, postoperative adjuvant therapy was not provided after consultation with the patient. However, six months after the pneumonectomy, maintenance therapy with pembrolizumab was resumed at the patient's request, and the patient is still receiving treatment.

## Discussion

Esophagobronchial fistulas commonly result from direct invasion of tumors of the esophagus, lungs, or mediastinum and can cause serious respiratory problems. Although most cases occur in the terminal stages of esophageal cancer, it is also a serious complication that can occur during radical chemoradiotherapy for advanced lung cancer [1,3-5]. Nevertheless, there is currently no established treatment plan for this condition. In general, esophagobronchial fistulas can be treated using surgical stenting, esophagectomy, esophageal bypass, and myocutaneous valve surgery; recently, self-expandable plastic stents such as the over-the-scope clip have also been used [2,6]. However, indications for such procedures are still largely based on experience [7].

The present case is of great value because, to our knowledge, this is the first report of esophagobronchial fistula as a complication of one course of preoperative chemotherapy with pembrolizumab in a patient with lung cancer who exhibited prominent tumor reduction after chemotherapy. The immunoinhibitor pembrolizumab has been recently recommended as a monotherapy or part of combination therapy for advanced non-small cell lung cancer with programmed cell death-ligand 1 positivity [8,9]. Furthermore, an undergoing analysis suggested that preoperative chemotherapy with pembrolizumab may be an early curative therapy that may benefit long-term tumor control. Considering the current evidence, reporting the occurrence of an esophagobronchial fistula during preoperative chemotherapy with pembrolizumab for lung cancer, as in this case, is important to consider adverse events.

Moreover, sharing a radical surgical approach for esophagobronchial fistula that occurred in this case is valuable, as only a few studies have reported on the surgical treatment of esophagobronchial fistulas. In the present case, bronchoscopy and upper endoscopy revealed a 2 cm fistula approximately 14 cm below the vocal cords, and stenting or over-the-scope clipping was considered difficult based on the diameter of the fistula [3,5]. The patient was in good condition, and the tumor had shrunk significantly after one course of preoperative chemotherapy. Therefore, a multidisciplinary conference on respiratory medicine and respiratory, esophageal, gastroenterology, and plastic surgery departments selected radical surgical resection as the treatment of choice.

In this case, the patient had a large esophagobronchial fistula, which was treated by suturing the fistula and filling it with stapedial valvuloplasty. During the procedure, a J-shaped incision was made in the lateral thoracic region, the subcutaneous tissue was dissected, and the entire border of the vastus lateralis muscle was reached to create a penile vastus valve. Subsequently, indocyanine green was administered and blood flow was confirmed using a PDE-neo camera. The high adhesions were removed using electrocautery, and the fistula was reached. The fistula was directly sutured using 4-0 PDS II with five stitches to the surrounding tissue. To fix the fistula, fibrin glue was applied, and suture ligation of the distal gastrocnemius valve with the surrounding tissue was performed. Esophagectomy and two-stage esophageal reconstruction were performed, and the patient was able to eat postoperatively. Thus, we believe that this series of surgical approaches in a rare case is valuable.

## Conclusions

We report a rare case of esophagobronchial fistula that developed during preoperative chemotherapy with pembrolizumab for lung cancer. The fistula was successfully treated by suturing and filling it with a distal hyoid valve. A multidisciplinary surgical approach should be considered in patients who are in good general condition and considered curable.
